# Urinary Phosphate and Subclinical Atherosclerosis: The AWHS Study

**DOI:** 10.3390/nu16162780

**Published:** 2024-08-20

**Authors:** Carolina Torrijo-Belanche, Belén Moreno-Franco, Martín Laclaustra, Sofía Gimeno-Ruiz, Naiara Calvo-Galiano, Jimena Rey-García, Pilar Guallar-Castillón

**Affiliations:** 1 Department of Preventive Medicine and Public Health, Universidad de Zaragoza, 50009 Zaragoza, Spain; carolinatorrijob@gmail.com; 2 Instituto de Investigación Sanitaria Aragón, Hospital Universitario Miguel Servet, 50009 Zaragoza, Spain; martin.laclaustra@unizar.es (M.L.); ncalvo@unizar.es (N.C.-G.); 3 CIBERCV (CIBER de Enfermedades Cardiovasculares), 28029 Madrid, Spain; 4 Department of Medicine, Psychiatry and Dermatology, Universidad de Zaragoza, 50009 Zaragoza, Spain; 5 Veterinary School, Universidad de Zaragoza, 50013 Zaragoza, Spain; gimenoruizsofia@gmail.com; 6 Internal Medicine Department, Ramón y Cajal University Hospital, Ramón y Cajal Health Research Institute, 28034 Madrid, Spain; jimena.reygarcia@gmail.com; 7 Department of Preventive Medicine and Public Health, School of Medicine, Universidad Autónoma de Madrid, 28029 Madrid, Spain; mpilar.guallar@uam.es; 8 CIBERESP (CIBER de Epidemiología y Salud Pública), 28029 Madrid, Spain; 9 IMDEA-Food Institute, CEIUAM+CSIC, Carretera de Cantoblanco 8, 28049 Madrid, Spain

**Keywords:** urinary phosphate, atherosclerosis, coronary arteries, carotid arteries, femoral arteries

## Abstract

(1) Background: Atherosclerosis is a leading cause of vascular death worldwide. High urinary phosphate has recently been identified as a cardiovascular risk factor, but its role has not been fully established. The aim of this study was to investigate the association between urinary phosphate and subclinical atherosclerosis in the carotid, femoral as well as coronary territories; (2) Methods: We performed a cross-sectional analysis of a sample of 1169 middle-aged men, aged 50.9 years (SD 3.7), without previous cardiovascular disease, belonging to the Aragon Workers Health Study (AWHS). Urinary phosphate was analyzed in urine samples using the Fiske-Subbarow method. The presence of carotid plaque and femoral plaque was assessed by ultrasound and coronary artery calcium score (CACS) by computed tomography. Demographic, anthropometric and clinical data were collected at annual medical examinations. Logistic regression models were used to estimate the prevalence of adjusted atherosclerosis in the different vascular arteries; (3) Results: A significant inverse association was observed between urinary phosphate and subclinical atherosclerosis in the carotid [OR 95% CI 0.69 (0.49–0.99)] and coronary (CACS > 200) [OR 95% CI 0.46 (0.23–0.88)] arteries; however, no statistically significant association was found between urinary phosphate and the presence of atheroma plaques in the femoral territory [OR 1.02 (0.72–1.45)]; (4) Conclusions: In middle-aged men, a higher urinary phosphate concentration is associated with a lower prevalence of subclinical carotid and coronary atherosclerosis compared with those with a lower urinary phosphate concentration.

## 1. Introduction

Atherosclerosis is a chronic progressive inflammatory disease of the arteries, mainly characterized by the accumulation of low-density lipoprotein (LDL-c), remnant lipoprotein particles, and other substances in the arteries [1,2]. Its exact incidence is difficult to determine because the condition is predominantly asymptomatic, with a latency of many years [3], with ischemic heart disease, ischemic stroke, and peripheral arterial disease being its main clinical manifestations [1]. Atherosclerosis is currently one of the leading causes of vascular death worldwide [4]. The onset and development of this pathology can be attributed to various traditional, genetic, and other factors related to cardiovascular disease (CVD) [5]. Large observational studies have identified many modifiable risk factors, and the causal relevance of some risk factors is now well established [6,7,8]. However, new risk factors, such as urinary phosphate concentration, continue to be investigated [9,10,11].

Phosphate homeostasis is essential for several physiological functions, such as bone formation [12,13,14,15], and a small percentage of phosphate is found in the extracellular compartment [12]. The body obtains this mineral from food, both in its natural form (organic phosphorus) and in salts added to food as additives (inorganic phosphorus) [16]. It has been suggested that a diet rich in phosphorus-based additives may have a negative impact on cardiovascular health, which may be one of the mechanisms underlying the apparent association between ultra-processed foods (UPF) and CVD [9].

Higher urinary phosphate concentrations have recently been associated with an increased risk of CVD [9,10,11]. In addition, some studies have suggested that urinary phosphate may have predictive value for all-cause and CVD mortality [10,17]. We hypothesize that higher urinary phosphate concentrations are positively associated with subclinical atherosclerosis. The aim of this study was to examine the association between urinary phosphate and subluminal atherosclerosis, including the presence of carotid and femoral artery plaques and coronary calcium, in a sample of middle-aged subjects from the Aragon Workers Health Study (AWHS).

## 2. Participants and Methods

The AWHS is a prospective cohort designed to assess the trajectories of traditional and emerging CVD risk factors and their association with the prevalence and progression of metabolic abnormalities and subclinical atherosclerosis in middle-aged workers at an automobile assembly plant who were free of clinical CVD and chronic kidney disease at baseline and recruited at an annual baseline examination between 2009 and 2010. The AWHS design and data collection methods have been described in detail previously [18,19]. From 2011 to 2014, participants aged 39 to 59 years were invited to undergo non-invasive imaging of subclinical atherosclerosis, complete dietary and lifestyle questionnaires, and provide blood and urinary samples. Only participants with information on atherosclerosis and urine samples were included. Women were excluded due to their small number (*n* = 68), and the final sample comprised 1169 men. The study was approved by the Ethics Committee for Clinical Research of the Institutional Review Board of Aragon. Written informed consent was obtained from all participants in the study.

### 2.1. Data Collection

#### 2.1.1. Urine Phosphate Sample

At baseline, spot urinary samples were collected in 5 mL polypropylene tubes, frozen within 1–2 h of collection, and were stored at −80 °C in the Aragon Biobank (Miguel Servet University Hospital, Zaragoza, Spain). In 2023, urinary phosphate concentrations were measured using the Fiske-Subbarow method for the in vitro determination of phosphorus in urine (QCA Kits) [20]. Urinary phosphorus was then divided by urinary creatinine.

#### 2.1.2. Subclinical Atherosclerosis Imaging

A Philips ultrasound system (IU22. Philips Healthcare, Bothell, WA, USA) was used to evaluate the occurrence of plaques in the carotid and femoral arteries. Ultrasound images were acquired with linear high-frequency two-dimensional probes (Philips Transducer L9-3, Philips Healthcare), using the Bioimage Study protocol for the carotid arteries [21] and an “ad hoc” designed protocol for the femoral arteries [22]. Examination of the carotid territory included four regions (terminal portion of common carotid, bulb, and initial portions of internal and external carotid arteries). The distance between the intima and media layers in a 2 cm segment proximal to the bifurcation was assessed by ultrasound in the femoral territory. A focal structure protruding > 0.5 mm into the lumen or reaching a thickness > 50% of the surrounding intima-media was defined as the presence of plaque in the carotid or femoral territories [22]. All measurements were taken at the end of the diastole (R-wave) and evaluated using electrocardiogram (ECG)-gated frames [23].

Coronary artery calcification (CAC) was obtained using non-contrast ECG-gated prospective acquisition with a 16-slice multidetector computed tomography scanner (Mx 8000 IDT 16, Philips Medical Systems, Best, The Netherlands). CAC was quantified using calcium scoring software (Workspace CT viewer, Philips Medical Systems) according to the Agatston method [24]. The Agatston method is a summed score of all coronary calcified lesions, taking into account both, the total area and the maximum density of coronary calcium. A high coronary artery calcium score (CACS) is a strong indicator of extensive cardiovascular disease with a significant amount of calcium deposits. The CACS is the reference standard and is the most commonly used score for coronary artery calcium in clinical practice [25]. CACS scores were divided into two categories (<200 and ≥200). A score of ≥200 indicates moderate to severe subclinical coronary atherosclerosis, which is associated with a clearly increased incidence of coronary heart disease [24].

### 2.2. Baseline Information on Covariates

Demographic, clinical as well as laboratory data were collected at the annual medical examination. These included age, medical history, current medication, body mass index (BMI) and waist circumference. Participants were categorized according to their smoking status as ever-smokers if they reported having smoked in the past year, or if they had smoked at least 50 cigarettes in their lifetime (but not in the past year), and as never-smokers. Physical examination was assessed using a validated Spanish version of the engagement in physical activity used in the Nurses’ Health Study and the Health Professionals’ Follow-up Study [26]. Arterial blood pressure was measured after a 5 min rest period using a digital sphygmomanometer (OMRON M10-IT. Healthcare Co., Ltd., Kyoto, Japan), recording the mean of three consecutive automatic readings. Measurements of the laboratory were performed on fasting blood samples (>8 h). Triglycerides, total cholesterol, high-density lipoprotein cholesterol (HDL-c), and serum glucose were determined by enzymatic analysis (ILAB 650 analyzer, Bedford, MA, USA). Non-HDL-c was calculated by subtracting HDL-c from total cholesterol. LDL-c was calculated using the Friedewald formula when triglyceride levels were <400 mg/dL [27]. Hypertension was defined as systolic blood pressure (≥140 mmHg), diastolic blood pressure (≥90 mmHg) or self-reported antihypertensive medication use [28]. Dyslipidemia was defined as total cholesterol ≥ 240 mg/dL, LDL-c ≥ 160 mg/dL, HDL-c < 40 mg/dL or self-reported lipid-lowering medication use [29]. Diabetes was defined as fasting serum glucose ≥ 126 mg/dL or self-reported glucose-lowering medication use [28].

### 2.3. Statistical Analysis

Participants were categorized into urinary phosphate concentration quartiles. Associations of urinary phosphate with subclinical atherosclerosis were examined using logistic regression models, adjusting for covariates. Odds ratios (ORs) and their 95% confidence intervals (CI) were calculated. Tests for linear trend (*p*-trend) across quartiles were calculated by taking the median for each quartile and modeling it as a continuous variable. Models were adjusted for age, BMI, smoking status, physical activity performed (total metabolic equivalents h/week), hypertension, dyslipidemia, and diabetes. Analyses were performed using R statistical software (ver. 4.1.3), and *p*-values below 0.05 were considered statistically significant.

## 3. Results

The mean age of the 1169 men included was 50.9 ± 3.7 years. Compared with those in the lowest quartile of urinary phosphate concentration, those in the highest quartile had higher BMI, waist circumference, and HDL-c levels (Table 1). The mean of urinary phosphate concentration divided by urinary creatinine was 0.6236 ± 0.6 mg/mg creatinine.

At least one carotid plaque was present in 443 participants (37.9%). The odds of having at least one carotid plaque were 0.69 (95% CI: 0.49, 0.99, *p*-trend 0.012) times lower in those in the highest quartile of urinary phosphate concentration than in those in the lowest quartile (Table 2).

At least one femoral plaque was present in 678 participants (58.0%). We observed no significant association between different concentrations of urinary phosphate excretion and the presence of subclinical atherosclerosis in the femoral arteries (fully adjusted OR 1.02; 95% CI: 0.72, 1.44, *p*-trend 0.567) (Table 2).

Any atherosclerotic plaque was present in 808 participants (69.1%). No significant association was found between the highest quartile of urinary phosphate concentration compared with the lowest quartile and the likelihood of having at least one atherosclerotic plaque in the femoral or carotid arteries (fully adjusted OR 0.91; 95% CI: 0.62, 1.33, *p*-trend 0.666) (Table 2).

A CACS > 200 was observed in 80 participants (6.8%). The odds of having a CACS > 200 were 0.46 (95% CI: 0.23, 0.88, *p*-trend 0.009) times lower in those in the highest quartile of urinary phosphate concentration than in those in the lowest quartile (Table 2).

## 4. Discussion

The results of our study suggest an inverse association between urinary phosphate concentration and subclinical atherosclerosis in the carotid and coronary arteries in otherwise healthy middle-aged men; however, no statistically significant association was found between urinary phosphate concentration and the presence of plaques in the femoral territory, although a slight U-shaped trend was observed. These results are interesting because urinary phosphorus is a marker of UPF consumption, and phosphorus has been postulated as a possible mechanism favoring the presence of subclinical atherosclerosis.

This is the first epidemiological study to examine the association between urinary phosphate and early subclinical atherosclerosis. Two previous articles have examined the relationship between urinary phosphate and incident CVD, with heterogeneous results. The study by Palomino et al. examined 24-h urinary phosphate excretion and the risk of cardiovascular events and found that participants in the highest tertile of urinary phosphate had a 30% lower risk of CVD compared with those in the lowest tertile. This study also showed that every 300 mg of urinary phosphate excreted per day was associated with a 17% lower CVD risk [30]. However, a recent study by Donat-Vargas et al., conducted in women only, showed an association between urinary phosphate concentrations and an increased risk of cardiovascular events. This association was stronger for acute myocardial infarction [HR 1.93 (1.02, 3.64; *p*-trend 0.061)] and weaker for stroke [HR 1.43 (0.86, 2.36; *p*-trend 0.178)] [9]. In contrast, this study found no association between the incidence of CVD or stroke and estimated dietary phosphorus intake, which was inversely associated with acute myocardial infarction [HR 0.55 (0.30, 1.00; *p*-trend 0.049)] [9]. For CVD mortality, there are only two published studies, both of which found no significant associations between total urinary phosphate and CVD mortality [10,17].

Another study focused on the association between dietary phosphorus intake and CACS in participants without a history of CVD or chronic kidney disease. They found no association between dietary phosphorus intake and the prevalence of subclinical coronary heart disease [31]. Kawamura et al. had also shown that inorganic phosphorus intake was associated with a greater reduction in vascular endothelial function than organic phosphorus in healthy young men [32], and that endothelial dysfunction underlies the atherosclerotic process [32,33].

Our current findings do not support a positive association between urinary phosphate (a marker for inorganic phosphate, mainly from UPF consumption) and atherosclerosis, at least in men in the early stages of cardiovascular disease. Nevertheless, inorganic phosphate may still be associated with the occurrence of cardiovascular events and CVD mortality in people with more advanced stages of the disease.

Phosphorus metabolism involves a variety of factors that influence both its intestinal absorption and urinary excretion. Some studies suggest that urinary excretion of this mineral does not correlate with total phosphorus intake [32,34]. However, other studies show that oral phosphorus intake is balanced by urinary and fecal phosphate excretion [35], and that urinary phosphate increases with the relative amount of inorganic phosphorus ingested, suggesting that urinary phosphate is a biomarker of inorganic phosphorus intake [32,36].

Almost all foods contain phosphorus; however, its absorption depends on the type of phosphorus present in these products (organic or inorganic) [32], with an average daily intake ranging from 700 to 2000 mg [35,36]. Inorganic phosphorus represents an important source of this mineral, and its absorption is highly efficient in healthy people, approaching close to 100% [37]. This type of phosphorus is the main component of many additives and preservatives, which have different purposes, such as prolonging shelf life, improving color, enhancing flavor, or retaining moisture [38,39]. Moreover, it is worth noting that the concentration of inorganic phosphorus in food additives is considered a health risk due to its increasing use and disproportionately high levels compared to organic phosphorus [38].

The other source of dietary phosphorus is organic phosphorus, which is present in natural protein-rich foods (animal as well as plant). The main sources of organic phosphorus from animals are meat, poultry, fish, dairy products, and eggs [40]. It is located in intracellular compartments and is easily hydrolyzed and absorbed, with intestinal absorption ranging from 50% to 70% [32,36,41]. In contrast, organic phosphorus from plants is mainly in the form of phytic acid or phytate and is present in high concentrations in some plant seeds, nuts as well as legumes [40]. Humans do not express the phytase-degrading enzyme, so its bioavailability is relatively low, and only 30 to 50% is absorbed [36,37,41].

In addition, phosphorus absorption is influenced by factors other than total phosphorus intake and its bioavailability, such as food preparation techniques, 1,25-(OH)2 vitamin D levels, and the presence or absence of compounds that can bind to phosphorus and inhibit its gastrointestinal absorption (such as calcium, aluminum, nicotinic acid, etc.) [32,35,37,41]. After absorption, phosphorus is transported through the cell membranes in the form of phosphate and is stored mainly in the bones [32,35]. Although it is also found in other organs such as blood, serum phosphate levels constitute < 1% of the total phosphorus reserves of the body [32].

In relation to renal phosphate excretion, it varies between 600 and 1500 mg/day, indicating that 75% to 85% of the daily filtrate is reabsorbed by the renal tubules. This urinary reabsorption is regulated by hormonal and metabolic factors such as phosphorus intake, 1,25-(OH)2-vitamin D, dopamine, or estrogen [35]. In addition, urinary phosphate as a urinary biomarker has been shown to have fair to excellent reproducibility (intraclass correlation coefficient 0.4), with minimal variation depending on sampling interval, frequency, and other variables [42].

Inorganic phosphorus-based additives and preservatives are present in a wide variety of foods to improve certain product properties, particularly in UPF [43]. The global increase in the consumption of UPF over the last century [44,45] has raised concerns that chronic high phosphate intakes may have adverse health effects [46], such as the development of early subclinical atherosclerosis.

Phosphorus additives are often present in UPF in the form of phosphoric acid, phosphates, or poly-phosphates. In Europe they are used as acidity regulators (E338–E343) and as emulsifiers and thickeners (E442, E450–E452, E544–E545). These additives are used for a variety of purposes, such as improving flavor, extending shelf life, regulating acidity, retaining moisture, or stabilizing frozen foods [16,41,47]. In addition, there are currently no regulations at the European level that require the inorganic phosphorus content of a food to be included in nutrition labeling, so food manufacturers are not obliged to provide this information [16,48,49]. Similarly, there are no established limits for the addition of phosphorus-containing preservatives [41]; only a scientific opinion published by the European Food Safety Authority (EFSA) in 2019 established an Acceptable Daily Intake (ADI) of 40 mg/kg body weight, considering both phosphorus from natural sources and from food additives [16].

It is estimated that phosphorus-containing additives contribute between 250 and 1000 mg to the total daily intake of phosphorus [46,50]. In addition, the use of phosphate additives is increasing [37,51,52]. This information is alarming because inorganic phosphorus from food additives has a greater effect than organic phosphorus on vascular endothelial function in healthy young men [32], so a high intake of inorganic phosphorus should now be considered a cardiovascular risk factor. However, inorganic phosphate content has not been included in previous studies to estimate intakes from nutrient databases because it is not feasible to do so as the added amount of food additives is not declared on food labels [32]. As a result, it is very difficult to determine the amount of phosphorus ingested and labeling of these substances is needed so that both healthy individuals and those with chronic kidney disease can make appropriate food choices [48]. Also, recent studies such as the one published in 2022 by Fulgoni et al. suggest that their disparate results on natural and added phosphorus intake support the need to update current food composition databases to provide accurate knowledge of dietary phosphorus intake (natural and added) [53].

Our study has several limitations. First, it is a cross-sectional analysis. Second, we have only one urinary phosphate measurement in the first morning spot urine, which may account for short-term changes in exposure [54,55]. It is preferable to collect 24-h urine samples over 2 to 3 days, which provide better reproducibility [42]. Finally, we cannot exclude the presence of residual confounding. For example, BMI was not homogeneously distributed between urinary phosphate quartiles, and some residual confounding may remain after adjustment. On the other hand, our study has valuable strengths. First, it includes the collection of clinical and imaging data from three different arterial territories according to strict protocols. Second, the participants in this male sample had a defined age range. This homogeneous age profile and the restriction to men helped to control for the most important confounding factors. The analyses were also controlled for the main cardiovascular confounders. In addition, we adjusted urinary phosphate for creatinine, as its concentration accounts for intra-individual variability [56,57].

## 5. Conclusions

In middle-aged men, a higher urinary phosphate concentration is associated with a lower prevalence of subclinical coronary and carotid atherosclerosis compared with those with a lower urinary phosphate concentration. Our results do not support a positive association between urinary phosphate concentration and prevalent atherosclerotic disease in middle-aged men without previous cardiovascular disease.

## Figures and Tables

**Table 1:** Baseline characteristics of the participants in the AWHS study by quartiles of urinary phosphate (N = 1169).

		Q1	Q2	Q3	Q4	
		<0.305	0.305–<0.398	0.398–<0.626	≥0.626	
N = 1.169	Mean	*n* = 294	*n* = 291	*n* = 292	*n* = 292	*p*-Trend
Age, years	50.9 (3.7)	51.1 (3.6)	50.9 (3.7)	51.2 (3.5)	50.4 (3.9)	0.095
BMI, kg/m^2^	27.6 (3.3)	27.3 (3.4)	27.4 (3.4)	28.0 (3.4)	27.9 (3.0)	0.017
Waist circumference, cm	97.6 (8.9)	96.8 (8.4)	96.4 (8.5)	99.0 (9.5)	98.4 (8.7)	0.001
Ever-smokers, %	76.8 [898]	76.1 [223]	75.7 [221]	80.5 [235]	75.0 [219]	0.375
Physical activity, total METs-h/wk	35.3 (21.5)	34.4 (21.5)	35.3 (21.2)	34.0 (20.9)	37.7 (22.1)	0.162
Systolic blood pressure, mm Hg	126.2 (13.8)	126.0 (13.4)	125.7 (13.7)	127.2 (14.5)	125.9 (13.6)	0.536
Diastolic blood pressure, mm Hg	83.5 (9.3)	82.7 (9.1)	83.2 (8.8)	84.5 (9.8)	83.4 (9.4)	0.138
Total cholesterol, mg/dL	223.7 (36.5)	223.8 (38.1)	224.4 (35.2)	222.2 (36.3)	224.4 (36.6)	0.862
HDL-c, mg/dL	52.5 (11.3)	50.8 (11.3)	53.2 (11.5)	52.9 (11.6)	53.0 (10.8)	0.031
Non-HDL-c, mg/dL	171.2 (35.3)	173.0 (37.1)	171.2 (33.4)	169.2 (35.2)	171.4 (35.5)	0.633
LDL-c, mg/dL	141.1 (31.8)	141.9 (33.3)	141.9 (30.5)	139.1 (31.8)	141.5 (31.7)	0.674
Triglycerides, mg/dL	156.4 (101.0)	167.0 (130.3)	151.4 (90.4)	152.4 (83.6)	154.7 (92.6)	0.211
Fasting glucose, mg/dL	99.2 (18.0)	98.1 (19.1)	98.8 (18.2)	100.0 (19.5)	99.9 (14.8)	0.539
Hypertension, %	39.4 [461]	40.6 [119]	39.0 [114]	42.1 [123]	36.0 [105]	0.465
Dyslipidemia, %	51.0 [596]	53.9 [158]	51.7 [151]	51.0 [149]	47.3 [138]	0.441
Diabetes, %	5.6 [65]	7.5 [22]	4.8 [14]	5.1 [15]	4.8 [14]	0.440

BMI: body mass index; HDL-c: high-density lipoprotein cholesterol; Non-HDL-c: non-high-density lipoprotein cholesterol; LDL-c: low-density lipoprotein cholesterol. Values are mean (SD) or % [number].

**Table 2:** Association between urinary phosphate and the presence of plaques in the AWHS study.

	Q1	Q2	Q3	Q4	
	<0.305	0.305–<0.398	0.398–<0.626	≥0.626	
N = 1169	*n* = 294	*n* = 291	*n* = 292	*n* = 292	*p*-Trend
Carotid plaques, % (*n*)	44.0% (*n* = 129)	39.7% (*n* = 116)	34.2% (*n* = 100)	33.6% (*n* = 98)	
Aged-Adjusted	Ref.	0.85 (0.61, 1.19)	0.64 (0.46, 0.91)	0.68 (0.48, 0.95)	0.008
Multivariable-Adjusted	Ref.	0.86 (0.61, 1.21)	0.63 (0.44, 0.89)	0.69 (0.49, 0.99)	0.012
Femoral plaques, % (*n*)	61.1% (*n* = 179)	52.1% (*n* = 152)	59.9% (*n* = 175)	58.9% (*n* = 172)	
Aged-Adjusted	Ref.	0.70 (0.50, 0.97)	0.94 (0.67, 1.32)	0.97 (0.69, 1.35)	0.698
Multivariable-Adjusted	Ref.	0.69 (0.49, 0.98)	0.91 (0.64, 1.29)	1.02 (0.72, 1.45)	0.567
Any atherosclerosis, % (*n*)	73.4% (*n* = 215)	65.8% (*n* = 192)	67.8% (*n* = 198)	69.5% (*n* = 203)	
Age-Adjusted	Ref.	0.70 (0.49, 1.00)	0.75 (0.52, 1.08)	0.88 (0.61, 1.27)	0.615
Multivariable-Adjusted	Ref.	0.71 (0.49, 1.02)	0.71 (0.49, 1.03)	0.91 (0.62, 1.33)	0.666
CACS > 200	11.6% (*n* = 34)	5.8% (*n* = 17)	4.8% (*n* = 14)	5.1% (*n* = 15)	
Aged-Adjusted	Ref.	0.47 (0.25, 0.85)	0.37 (0.19, 0.70)	0.44 (0.23, 0.83)	0.004
Multivariable-Adjusted	Ref.	0.49 (0.25, 0.91)	0.40 (0.20, 0.76)	0.46 (0.23, 0.88)	0.009

CACS, coronary artery calcium score; n, number of subjects with plaques. Multivariable adjusted for age, BMI, smoking status, physical activity, hypertension, dyslipidemia, and diabetes.

## Data Availability

The data presented in this study are available on request from the corresponding author. The data are not public due to ethical reasons.
